# Core/Sheath-Structured Composite Nanofibers Containing Cinnamon Oil: Their Antibacterial and Antifungal Properties and Acaricidal Effect against House Dust Mites

**DOI:** 10.3390/polym12010243

**Published:** 2020-01-20

**Authors:** Yoonwon Jung, Hyukjoo Yang, In-Yong Lee, Tai-Soon Yong, Seungsin Lee

**Affiliations:** 1 Department of Clothing and Textiles, Yonsei University, Seoul 03722, Korea; 2 Department of Environmental Medical Biology, Institute of Tropical Medicine, College of Medicine, Yonsei University, Seoul 03722, Korea

**Keywords:** emulsion electrospinning, nanofiber, core/sheath structure, cinnamon oil, acaricidal effect, house dust mite, antibacterial property, antifungal property

## Abstract

This study aimed to fabricate core/sheath-structured composite nanofibers containing cinnamon oil by emulsion electrospinning and to investigate their acaricidal effect on house dust mites as well as their antibacterial and antifungal properties in relation to cinnamon oil concentration in the nanofibers. An oil-in-water emulsion, which comprised cinnamon oil and poly(vinyl alcohol) solution as oil and water phases, respectively, was used to prepare core/sheath-structured nanofibers. The morphology and the inner structure of the electrospun nanofibers were observed by scanning electron microscopy and confocal laser scanning microscopy. Core/sheath-structured nanofibers containing cinnamon oil were successfully prepared by emulsion electrospinning. The composite nanofibers prepared from an emulsion containing 20 wt% of cinnamon oil exhibited a strong acaricidal effect against house dust mites (*Dermatophagoides farinae*). The composite nanofibers fabricated from an emulsion containing 4.29 wt% of cinnamon oil showed excellent antimicrobial effects against *Staphylococcus aureus* and a series of fungi that can trigger respiratory- and skin-related diseases. The release profile of cinnamon oil from the core/sheath-structured nanofibers showed a continuous release of functional ingredients over 28 days. Our findings demonstrate that the use of such fibrous structures could be a promising approach for delivering naturally derived bioactive agents in a controlled way.

## 1. Introduction

There has been a recent increase in interest in the level of hygiene in indoor environments, prompted by the fact that people are spending more time indoors and by changes in residential environments. In this context, studies related to the development of environmentally friendly home textile products using natural extracts have received considerable attention.

Indoor pollutants such as allergens, endotoxins, mycotoxins separated from house dust mites, bacteria, and fungi serve as antigens that induce environmental diseases including asthma, allergic rhinitis, and atopic dermatitis [1]. In particular, changes in human living environments, including central heating, sealed windows, and fitted carpets have led to an increase in normal indoor temperature and humidity levels. It is difficult to launder certain home textile products such as sofas, carpets, and blankets, thereby creating an environment conducive to the growth of house dust mites. Therefore, it is crucial to prevent the spread and growth of house dust mites by using chemical treatments, including treatments that contain acaricides, or by physical treatments, including washing or cleaning home textiles [2,3].

Although currently favored synthetic acaricides can control house dust mites, the repeated use of these chemicals has led to the development of resistance, leading to deleterious effects on the environment and human health. There is increasing interest in some essential oils that possess antibacterial, antifungal, antiviral, and acaricidal as well as anti-inflammatory properties [4,5,6,7,8]. Essential oils are volatile, natural compounds synthesized by aromatic plants as secondary metabolites [9]. Cinnamon bark essential oil is obtained from the bark of the Cinnamomum species, with cinnamaldehyde being its main component. Cinnamaldehyde is well known for its efficient acaricidal effect on house dust mites, as well as its antibacterial, anti-inflammatory, and antioxidant properties [10].

Emulsion electrospinning is a novel method for fabricating core/sheath-structured composite nanofibers with an ordinary single-nozzle electrospinning setup. The mechanism for transforming an emulsion to core/sheath-structured fibers involves the stretching of the jet and evaporation of solvents by the application of electrical charges on the emulsion droplet surface [11]. In this process, a water-insoluble oil phase assembles within a hydrophilic water-soluble polymer such as poly(vinyl alcohol) (PVA) [8,12]. The method is highly valued in biomedical applications, for example, in drug delivery systems that provide controlled release rates of drugs [13,14].

Cinnamon oil is known to be a natural insecticide because it is a mixture of biologically active substances, has a selective insecticidal and acaricidal effect only on target organisms, and has few side effects on the human body and the environment [4]. However, when cinnamon oil is dispersed in a liquid state on home textiles, it is difficult to impart continuous functionality to the textile fibers due to its volatile nature. Incorporating cinnamon oil in the core of nanofibers through emulsion electrospinning could create eco-friendly home textiles that can steadily release the functional components that suppress the growth of house dust mites and strains. Although the encapsulation of cinnamon oil in a nanofibrous film for application in wound dressing and active food packaging has been reported [15,16], the potential acaricidal effects of composite nanofibers against house dust mites have yet not been studied. Hence, in the present work, an attempt has been made to explore the possible acaricidal effects of composite nanofibers against house dust mites and their antimicrobial effects against bacteria and a series of those fungi that trigger respiratory- and skin-related diseases in home textile applications.

This study aimed to fabricate core/sheath composite nanofibers containing cinnamon oil and PVA via emulsion electrospinning and to investigate their acaricidal effects against house dust mites, as well as their antimicrobial properties in relation to the cinnamon oil concentration in the nanofibers. The morphology and inner structure of the electrospun nanofibers were examined, and the release characteristics of cinnamon oil from the composite nanofibers were assessed over a 28-day period to examine the sustained release of functional ingredients over a period of time.

## 2. Materials and Methods

### 2.1. Materials

The essential cinnamon oil was supplied by Neumond (Raisting, Germany). The oil was 100% natural essential oil obtained through steam distillation of the bark of Cinnamomum trees growing in Sri Lanka. Poly(vinyl alcohol) (99% hydrolyzed, *M*_w_ = 89,000−98,000) was provided by Sigma Aldrich Co. (St. Louis, MO, USA). A nonionic surfactant, Tween 80, was supplied by Kao Co. (Tokyo, Japan). Tween 80, a surfactant having a hydrophilic–lipophilic balance value of 15, was used to stabilize oil-in-water (O/W) emulsions. Nile red (NR) and fluorescein isothiocyanate (FITC), which are fluorescent markers, were obtained from Sigma Aldrich Co. (St. Louis, MO, USA) and used for confocal laser scanning microscopy (CLSM) analysis.

### 2.2. Emulsion Preparation

An O/W emulsion composed of PVA solution as the water phase and cinnamon oil as the oil phase was prepared before electrospinning. First, PVA was dissolved in distilled water at 80 °C for 6 h to prepare PVA solutions. A certain amount of cinnamon oil and Tween 80 (surfactant) was added to the aqueous PVA solution (exact emulsion compositions are specified later), and the emulsion was then stirred at 1000 rpm for 1 h. The surfactant was added to decrease the interfacial tension between the aqueous phase and the oil phase so that the cinnamon oil could be well dispersed in the PVA solution as a spherical droplet.

In preliminary experiments, there was a significant difference between the emulsion concentrations that exhibited antimicrobial effects and acaricidal effects against house dust mites. That is, acaricidal effects against house dust mites were demonstrated with much higher added oil content than that required in the cinnamon oil concentrations to achieve antimicrobial effects. Therefore, based on the preliminary experiments, the emulsion was prepared in two concentration ranges. Table 1 and Table 2 show the concentration ranges of the emulsion to impart antimicrobial effects and the concentration ranges of the emulsion to impart acaricidal effects against house dust mites, respectively. The concentration ranges to impart antimicrobial activity were set to 10–14 wt% of PVA, 3.57–5 wt% of cinnamon oil, and 0.71–1 wt% of surfactant (Tween 80). For the concentration ranges to impart acaricidal effects against house dust mites, the PVA concentration was fixed to 12 wt% and the concentrations of cinnamon oil and surfactant (Tween 80) were set within the ranges 4.29–30 and 0.86–6 wt%, respectively. The concentrations of the oil and surfactant were fixed at a ratio of 5:1 (*w*/*w*) based on previous studies [8,12].

### 2.3. Emulsion Electrospinning

A vertical electrospinning setup with an ordinary single nozzle (NNC-ESP200R2, NanoNC Co., Seoul, Korea) was used for emulsion electrospinning. The setup consists of a syringe with a needle, a syringe pump, a high-voltage power supply, and a grounded collector. The O/W emulsion, comprising cinnamon oil and PVA solution, was loaded into the syringe, and an electrode was clipped to the needle. The syringe pump delivered emulsions at a constant feed rate ranging from 0.2 to 1.2 mL/h. When a high voltage of 25 kV was applied to the needle, an emulsion droplet at the needle tip was stretched into a jet forming a Taylor cone, and this electrical charge-induced jet ejected from the needle tip underwent rapid elongation and solvent evaporation. The needle gauges used were 23–25 (0.26–0.34 mm i.d.), and the tip-to-collector distance was maintained at 25 cm. Nanocomposite fibers were electrospun and deposited onto a substrate mounted on the grounded collector. The substrate fabric was 100% cotton fabric with a fabric weight of 170 g/m^2^ and a thickness of 0.3 mm. All specimens were stored in sealed plastic bags at room temperature until further analysis.

### 2.4. Fiber Morphology

The morphology of the electrospun nanofibers containing cinnamon oil was examined by field emission scanning electron microscopy (JSM-7800F, JEOL Ltd., Tokyo, Japan) to find the most suitable emulsion concentrations and electrospinning processing conditions for fabricating bead-free nanocomposite fibers. Before observing the morphology of the nanofibers, Pt coating was performed for 90 s on the surface of the fibers under vacuum conditions. Fiber diameters were measured from the SEM micrographs using image analysis software (ImageJ, National Institutes of Health, Bethesda, MD, USA). The average fiber diameter was determined by measuring the diameter of 30 random fibers from three different micrograph images.

To further characterize the morphology of the bicomponent fibers and visualize the distribution of cinnamon oil within the fibers, CLSM was used. NR, a selective fluorescent dyestuff with lipophilic properties, was used as a fluorescent marker to track the oil within the fibers for CLSM analysis. Cinnamon oil was labeled by dissolving 0.01 g/mL NR in the oil before electrospinning. Emulsions containing NR-labeled oils were electrospun, and the composite fibers were examined under a confocal laser scanning microscope (CLSM 700, Carl Zeiss, Oberkochen, Germany) to visualize the distribution of NR-labeled oils within the fibers. An excitation wavelength of 515 nm was used for the analysis. In addition, the aqueous PVA solution was stained with a water-soluble fluorescent dye, FITC, in order to visualize the PVA. FITC (1% (*w*/*w*) based on the weight of PVA) was reacted with an aqueous PVA solution in the dark and stirred for 48 h, prior to emulsification. Emulsions containing FITC-labeled PVA were electrospun, and the composite fibers were examined under a CLSM. An excitation wavelength of 492 nm was used for observing the FITC fluorescence.

### 2.5. Assessment of the Acaricidal Effect of Nanocomposite Fibers against House Dust Mites

**Figure 1:**
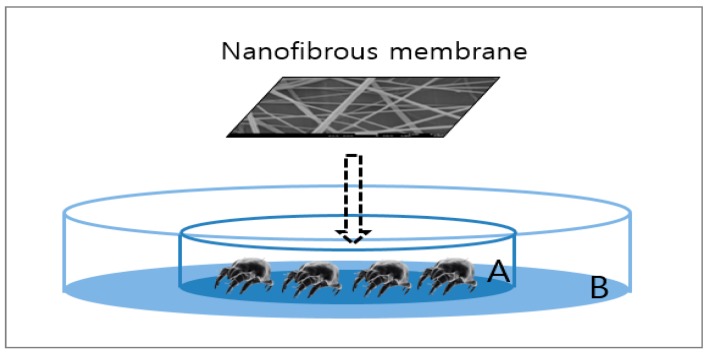
Experimental setup for assessing the acaricidal effects of nanofibrous membranes containing cinnamon oil on house dust mites.

The acaricidal effect of cinnamon oil-comprising nanocomposite fibers against house dust mites (*Dermatophagoides farinae*) was examined by the direct contact method. *Dermatophagoides farinae* is the dominant species of house dust mites in Korea and allergens separated from it can cause respiratory- and skin-related allergic diseases [17]. The experimental setup is depicted in Figure 1. Batches of 30 adult mites were placed on a piece of cotton gauze (30 mm × 30 mm), which was placed in the middle of petri dish A (50 mm diameter × 10 mm height). Then, the treated specimen (the composite nanofibrous membrane containing cinnamon oil) and a control specimen comprising only PVA were placed respectively on top of the cotton gauze. Petri dish A was placed in the middle of petri dish B (100 mm diameter × 15 mm height), and Vaseline was applied to the area of petri dish B outside petri dish A to prevent the mites from crawling out. Specimens were prepared with a web area density of 6.4 g/m^2^. The test was performed for 1 h under conditions of 25 ± 1 °C, 80% RH, which was similar to the environment where the mites were cultured. Mites were considered dead if they were flipped over with four legs stretched out and were no longer moving. The mortality rate of house dust mites was determined using the following formula:

(1) $$R\;\left(\%\right)\;=\frac{A-B}{A}\times 100$$

where R is the rate of house dust mite mortality, A is the number of house dust mites from the control specimens surviving after 1 h, and B is the number of house dust mites from the treated specimens surviving after 1 h.

### 2.6. Antibacterial Activity

The antibacterial activity of the nanofibrous membranes containing cinnamon oil was determined quantitatively by ASTM E 2149-13 (Standard Test Method for Determining the Antimicrobial Activity of Antimicrobial Agents under Dynamic Contact Conditions) [18]. Two representative microorganisms, *Staphylococcus aureus* (ATCC 6538, Gram-positive bacterium) and *Klebsiella pneumoniae* (ATCC 4352, Gram-negative bacterium), were used for the assessment. Specimens were prepared with a web area density of 9.0 g/m^2^.

To carry out antimicrobial assessments, the fibers should be stable in aqueous environments since the fibers must be immersed in an inoculated buffer solution during the test procedure. Thus, heat treatment was applied to the membranes before the antimicrobial evaluation to improve the aqueous stability of PVA-based nanofibrous membrane samples. PVA nanofibrous membranes containing cinnamon oil were heat-treated at 160 °C for 1 min to stabilize PVA composite nanofibers against dissolution in a buffer solution. The heat treatment conditions were chosen based on preliminary experimental trials.

For the antimicrobial evaluation, the specimens were placed in an inoculated buffer solution within a flask and were stirred with a shaker at a temperature of 37 ± 1 °C for 24 h. After incubation for another 24 h, the number of bacterial colonies was compared with the buffer solution (control) with inoculum only. The reduction rate of test organisms after contacting the treated specimen was determined using the following formula:

(2) $$R\;\left(\%\right)\;=\frac{A-B}{A}\times 100$$

where R is the reduction rate of the number of colonies, A is the number of bacterial colonies in the flask with inoculum only after 24 h, and B is the number of bacterial colonies in the flask containing the treated specimen after 24 h of contact time.

### 2.7. Antifungal Activity

The antifungal effect of the nanofibrous membranes containing cinnamon oil was examined qualitatively in accordance with ASTM G 21-15 (Standard Practice for Determining Resistance of Synthetic Polymeric Materials to Fungi) [19] on a series of those fungi that trigger respiratory- and skin-related diseases. The fungal species tested included *Aspergillus brasiliensis* (ATCC 9642), *Penicillium funiculosum* (ATCC 11797), *Chaetomium globosum* (ATCC 6205), *Blue fungus Trichoderma virens* (ATCC 9645), and *Aureobasidium pullulans* (ATCC 15233). Specimens were prepared with a web area density of 13.8 g/m^2^. The specimens were finely cut and placed on the medium, in which five fungi were inoculated, and the strain was cultured under conditions of 28 °C and 85% RH for 28 days. The criteria for the evaluation of antifungal activity were determined by visual observation of the fungal growth in five grades. The grading scale for this test is shown in Table 3. All the measurements were carried out in triplicate.

### 2.8. Cinnamon Oil Release Studies

The release profile of cinnamon oil from the nanocomposite fibers was examined over a 28-day period using a thermal extractor and thermodesorption gas chromatography with mass spectrometric detection (TDS-GC/MS). Specimens were prepared with a web area density of 9.0 g/m^2^. The release of cinnamon oil from the composite fibers was assessed five times during the testing period, i.e., immediately after electrospinning and 7, 14, 21, and 28 days after electrospinning. During the testing period, the specimens were stored under conditions of 20–25 °C and 30%–40% RH to simulate release in real-use situations.

Composite nanofibers were placed in a Tenax tube, and then air sampling was carried out using a thermal extractor (Gerstel, Mülheim an der Ruhr, Germany). Nitrogen (N_2_) was used as a carrier gas with a flow rate of 39 mL/min, and 1 L of gas was collected at 25 °C. The captured gas was analyzed by GC (Clarus 680, Perkin Elmer, Waltham, MA, USA) equipped with an Elite-5MS column (Perkin Elmer, Waltham, MA, USA) (60 m × 320 μm × 1.8 μm). The initial column temperature was maintained at 50 °C for 5 min and then increased at 5 °C/min until 220 °C; this temperature was held for 10 min. The temperature was raised to a final temperature of 250 °C at a rate of 10 °C/min and kept for 5 min when the final temperature was reached. The carrier gas used was helium at a flow rate of 1 mL/min. All the measurements were carried out in triplicate.

## 3. Results and Discussion

### 3.1. Fiber Morphology of Emulsion Electrospun Nanofibers

#### 3.1.1. Composite Nanofibers Containing Low Concentrations of Cinnamon Oil

**Figure 2:**
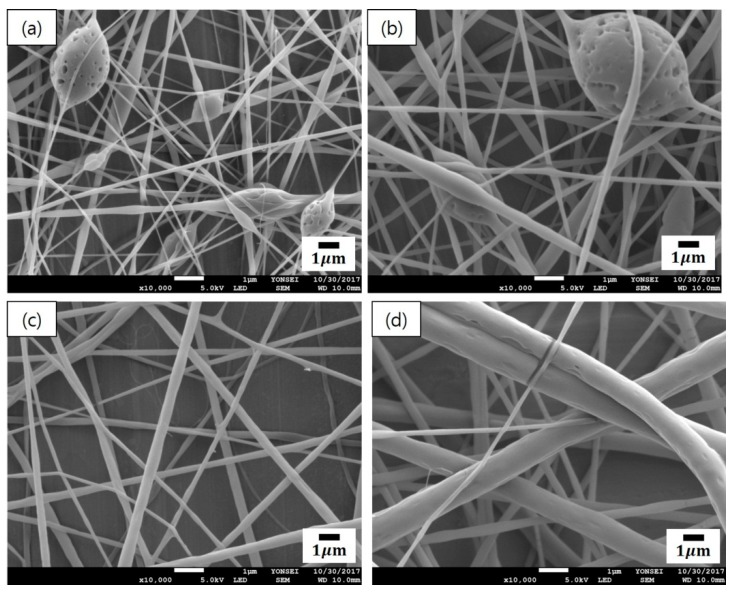
SEM images of cinnamon oil-containing poly(vinyl alcohol) (PVA) nanofibers produced from different emulsion formulations: (**a**) emulsion A (10 wt% of PVA, 3.57 wt% of oil, and 0.71 wt% of surfactant), (**b**) emulsion B (11 wt% of PVA, 3.93 wt% of oil, and 0.79 wt% of surfactant), (**c**) emulsion C (12 wt% of PVA, 4.29 wt% of oil, and 0.86 wt% of surfactant), and (**d**) emulsion D (13 wt% of PVA, 4.64 wt% of oil, and 0.93 wt% of surfactant).

Figure 2 presents the SEM micrographs of cinnamon oil-containing PVA nanofibers produced from different emulsion formulations (emulsions A–D in Table 1). In the range of 10–11 wt% of PVA concentration (emulsions A and B), many beads were observed on the fibers (Figure 2a,b). Emulsions A and B yielded beaded composite fibers with an average diameter of 166 ± 24 nm and an average diameter of 266 ± 57 nm, respectively. At the concentration of 12 wt% of PVA (emulsion C), beads disappeared and straight fibers were formed, which had relatively smooth surfaces and an average diameter of 299 ± 56 nm (Figure 2c). At the concentration of 13 wt% of PVA (emulsion D), the diameter of the fibers increased, and the fiber size distribution became broad (Figure 2d). Emulsion D yielded relatively straight composite fibers with an average diameter of 507 ± 152 nm. At the concentration of 14 wt% of PVA (emulsion E), the viscosity of the solution was so high that electrospinning was not possible. When the concentration of the polymer solution is low, beads are formed on the nanofibers due to the influence of the surface tension, which is the attraction between the solvent molecules in the polymer solution [20]. As the concentration of PVA increased, the number of beads decreased and the fiber diameter increased. This may be attributed to the increase in the entanglement between the polymer chains with increasing polymer concentration. These observations were consistent with previous reports [20,21]. As shown in Figure 2c, emulsion C (containing 12 wt% of PVA, 4.29 wt% of cinnamon oil, and 0.86 wt% of surfactant) yielded bead-free-shaped nanofibers with little deviation in fiber size compared with the other concentrations. Thus, emulsion C was chosen to further examine the optimal processing conditions for producing uniform nanocomposite fibers.

**Figure 3:**
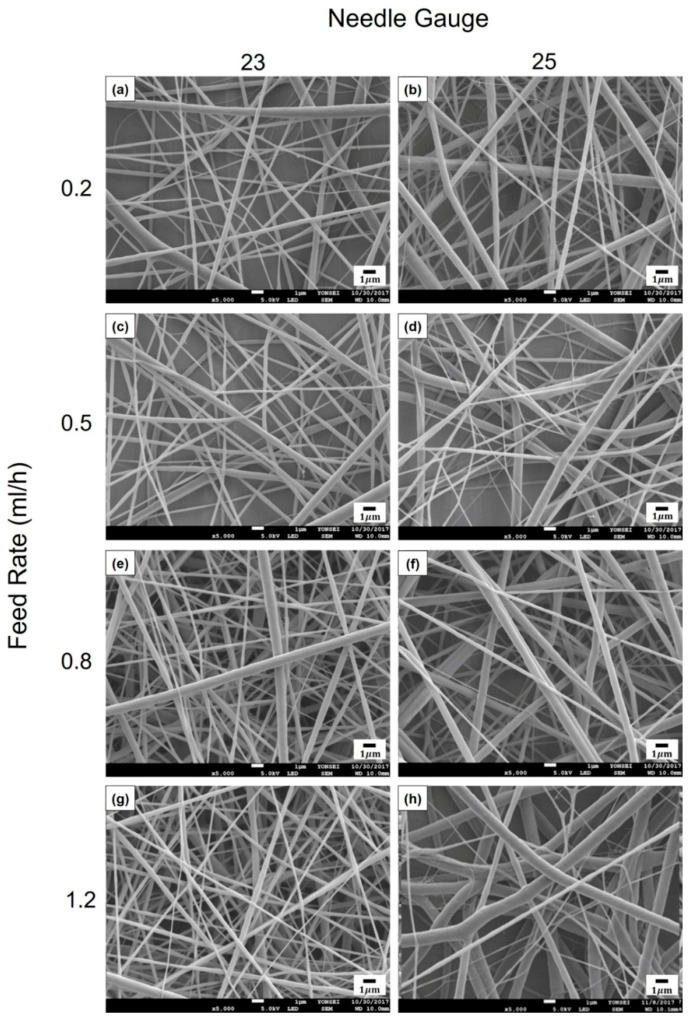
SEM images of cinnamon oil-containing PVA nanofibers produced under different processing conditions: (**a**) 0.2 mL/h, 23 G, (**b**) 0.2 mL/h, 25 G, (**c**) 0.5 mL/h, 23 G, (**d**) 0.5 mL/h, 25 G, (**e**) 0.8 mL/h, 23 G, (**f**) 0.8 mL/h, 25 G, (**g**) 1.2 mL/h, 23 G, and (**h**) 1.2 mL/h, 25 G. Applied voltage and tip-to-collector distance were maintained constant at 25 kV and 20 cm, respectively.

**Figure 4:**
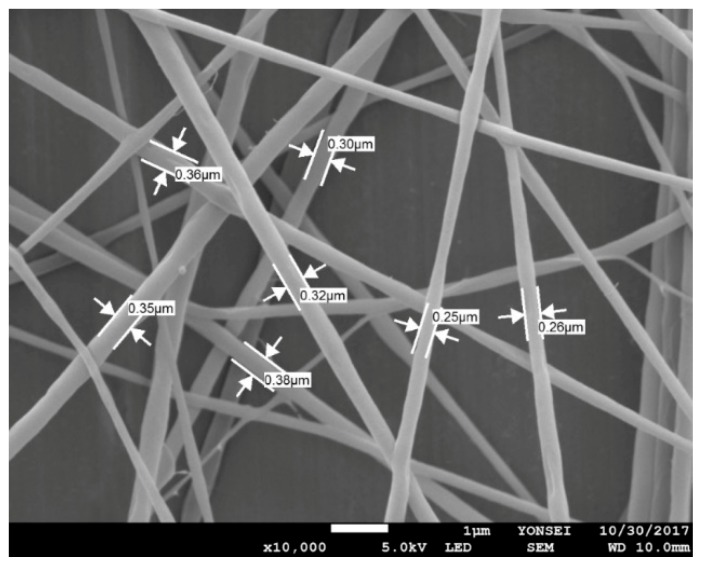
SEM image of cinnamon oil/PVA nanofiber webs from emulsion C (12 wt% of PVA, 4.29 wt% of cinnamon oil, and 0.86 wt% of surfactant) produced with a solution feed rate of 0.2 mL/h, a voltage of 25 kV, and a tip-to-collector distance of 20 cm through a 23-gauge needle.

For the emulsion formulation selected, electrospinning was conducted using different needle gauges under various solution feed rates. Figure 3 shows the morphology of composite nanofibers spun from emulsion C under various spinning conditions, including feed rates of 0.2–1.2 mL/h and needle gauges of 23–25. The voltage applied and tip-to-collector distance were set to 25 kV and 20 cm, respectively. As shown in Figure 3b,d,f,h, a 25-gauge needle yielded fiber size distributions that tended to be broader. Among the composite fibers obtained with the 23-gauge needle, straight fibers with less variation in fiber size were obtained at a feed rate of 0.2 mL/h (Figure 3a) compared with fibers produced under other conditions. Thus, the most suitable spinning conditions for fabricating smooth nanocomposite fibers with even fiber size distribution were found to include a solution feed rate of 0.2 mL/h, a voltage of 25 kV, and a tip-to-collector distance of 20 cm through a 23-gauge needle. Figure 4 presents the SEM micrograph of cinnamon oil-containing PVA nanofibers obtained from emulsion C (containing 12 wt% of PVA, 4.29 wt% of cinnamon oil, and 0.86 wt% of surfactant) under the aforementioned spinning conditions. These conditions produced bead-free fibers with an average diameter of 299 ± 56 nm.

**Figure 5:**
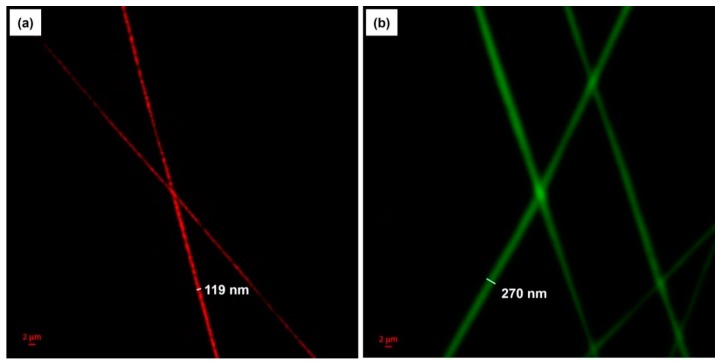
CLSM images of cinnamon oil/PVA nanofibers from emulsion C: (**a**) Cinnamon oil was stained by Nile red and viewed with excitation at 515 nm. (**b**) PVA was stained by FITC and viewed with excitation at 492 nm.

To further examine the morphology of the bicomponent fibers and visualize the distribution of cinnamon oil within the fibers, the nanocomposite fibers produced from emulsion C under the optimal conditions were characterized using CLSM. A fluorescent and lipophilic dye, NR, was used to label the non-fluorescent cinnamon oil. As presented in Figure 5a, the NR-labeled cinnamon oil was excited and emitted light at a wavelength of 515 nm, shown in red. In addition, FITC, a water-soluble fluorescent dye, was used to label the PVA to further verify the morphology of the bicomponent fibers. Figure 5b shows the FITC-labeled PVA sheath, viewed with green excitation at a wavelength of 492 nm. The CLSM images revealed that cinnamon oil was successfully embedded in the core of the fibers. The core/sheath-structured fibers have an overall diameter of 270 nm and a core diameter of 119 nm. It was reported that core/sheath structures are generated due to the stretching and coalescence of the emulsion during electrospinning [11]. This is associated with the rapid elongation and the differences in volatility between the two liquid phases. When the solvent of the polymer solution forming the continuous phase evaporates more rapidly, the viscosity of the continuous phase becomes greater than that of the dispersed phase. This causes the emulsion droplets to move inward during electrospinning and merge in the core of the fiber. Our CLSM images confirm the presence of well-aligned core/sheath structures of the cinnamon oil-loaded PVA nanofibers.

#### 3.1.2. Composite Nanofibers Containing High Concentrations of Cinnamon Oil

**Figure 6:**
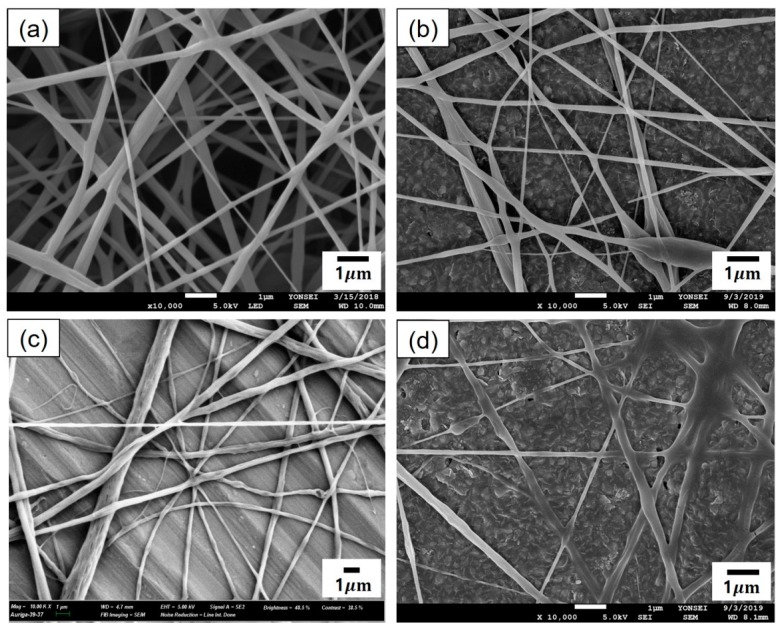
SEM images of cinnamon oil-containing PVA nanofibers produced from emulsion formulations containing high concentrations of cinnamon oil: (**a**) emulsion F (8 wt% of oil, 12 wt% of PVA, and 1.6 wt% of surfactant), (**b**) emulsion G (15 wt% of cinnamon oil, 12 wt% of PVA, and 3 wt% of surfactant), (**c**) emulsion H (20 wt% of cinnamon oil, 12 wt% of PVA, and 4 wt% of surfactant), and (**d**) emulsion I (30 wt% of cinnamon oil, 12 wt% of PVA, and 6 wt% of surfactant). Solution feed rate, applied voltage, needle gauge, and tip-to-collector distance were maintained constant at 0.2 mL/h, 25 kV, 23-gauge, and 20 cm, respectively.

For the emulsion formulations containing high concentrations of cinnamon oil (emulsions F–I in Table 2), electrospinning was conducted under the same spinning conditions as the optimal conditions determined for emulsion C (i.e., a solution feed rate of 0.2 mL/h, a voltage of 25 kV, a tip-to-collector distance of 20 cm, and a 23-gauge needle). Figure 6 shows the morphology of composite fibers electrospun from emulsions F–I. It is noteworthy that relatively uniform composite fibers were successfully fabricated from the emulsions with cinnamon oil concentrations of 8–30 wt%. The emulsions containing high concentrations of cinnamon oil yielded fibers with diameters greater than those of the nanofibers fabricated using low concentrations of cinnamon oil in the emulsion. As indicated in Figure 6, composite fibers with relatively smooth surfaces were obtained from emulsion H (Figure 6c) even with an increase in oil concentration of 20 wt% in the emulsion. Emulsion H (containing 12 wt% of PVA, 20 wt% of cinnamon oil, and 4 wt% of surfactant) yielded bead-free-shaped composite fibers with an average diameter of 526 ± 19 nm.

**Figure 7:**
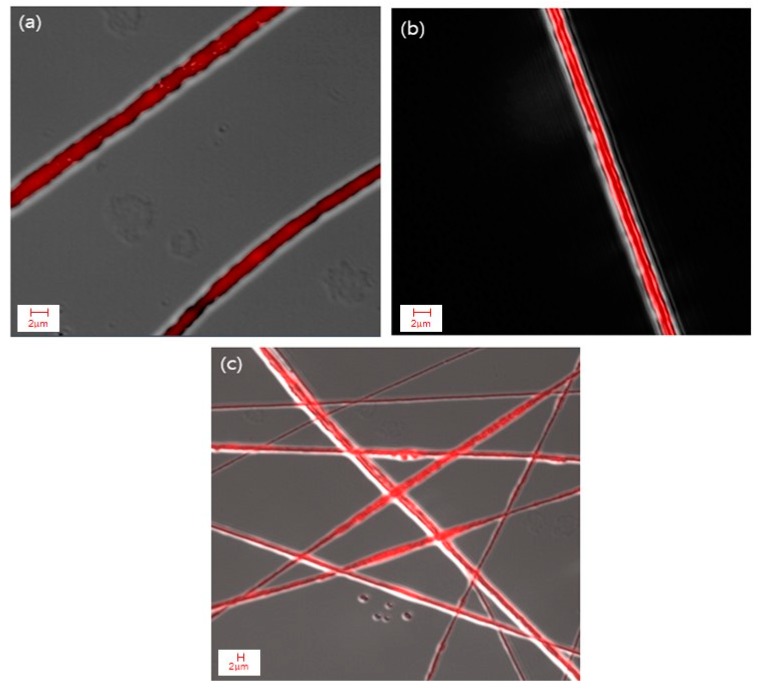
CLSM images of cinnamon oil/PVA nanofibers from emulsion H: (**a**–**c**) core/sheath structures of cinnamon oil/PVA nanofibers.

The composite fibers produced from emulsion H under the aforementioned spinning conditions were further examined using CLSM after labeling cinnamon oil with NR. Figure 7 shows that the core/sheath-structured fibers were successfully fabricated and the oil was distributed continuously throughout the fiber core even with an increase in the oil concentration of up to 20 wt% in the emulsion.

### 3.2. Acaricidal Effects of Core/Sheath-Structured Nanofibers Containing Cinnamon Oil against House Dust Mites

The acaricidal effects of composite nanofibers containing cinnamon oil against house dust mites (*Dermatophagoides farinae*) were investigated to identify oil concentrations that provide effective acaricidal effects against house dust mites. For this purpose, the direct contact method was used to assess the acaricidal activity of the composite nanofibers containing high concentrations of cinnamon oil. Moreover, to find out whether drying the composite nanofibers after electrospinning would affect the acaricidal activity, the assessment was conducted on membranes dried in a vacuum oven at 50 °C for 24 h after electrospinning as well as the as-spun membranes.

Table 4 presents the acaricidal effects of the composite membranes containing cinnamon oil against house dust mites at various oil concentrations. For the control samples that do not contain cinnamon oil, all 30 mites were alive after 1 h, showing no acaricidal effects against house dust mites. However, the as-spun nanofibrous membranes fabricated from the emulsions with cinnamon oil concentrations of 20 wt% (emulsion H) and 30 wt% (emulsion I) exhibited 100% mortality of house dust mites. All the mites died within 1 h after coming into direct contact with these as-spun composite nanofibrous membranes. Therefore, the as-spun nanofibers fabricated from emulsion H (containing 12 wt% of PVA, 20 wt% of cinnamon oil, and 4 wt% of surfactant) can be referred to as the optimum concentration to provide effective acaricidal effects against house dust mites for our experimental conditions. This finding is similar to the results of a previous study in which mites placed on a cloth sprinkled with cinnamaldehyde exhibited a >90% mortality rate within 1 h [22]. On the other hand, nanocomposite fibers dried after electrospinning did not exhibit any acaricidal effects against house dust mites.

Monoterpene is a major component of the cinnamon essential oil, and it is known for interfering with the nervous system of insects. The terpene components containing oxygenated chemical structures such as eugenol and cinnamaldehyde make cinnamon oil a highly biologically active material [23,24]. The insecticidal properties of these biologically active substances in essential oils are achieved by inhibiting nerve transmission in insects, but side effects are rare in mammals [25]. Therefore, nanocomposite fibers containing cinnamon oil could be used as a safe and harmless insecticidal material and replace synthetic acaricides, which present potential risks when continuously exposed to the human body.

### 3.3. Antibacterial Properties of Core/Sheath-Structured Nanofibers Containing Cinnamon Oil

Although various antimicrobial agents have been utilized to control microbial infections [26,27,28,29,30], synthetic biocides can cause skin irritation or allergy. Thus, natural plant-derived antimicrobial agents, which are environmentally friendly and non-toxic, have attracted much attention as a result of their potential for use in home textiles where textiles are in direct contact with human skin. In this study, the antibacterial activity of the nanofibrous membranes containing cinnamon oil was assessed to examine whether the nanocomposite fiber webs could be applied to home textiles to improve hygiene in indoor environments. Since cinnamon oil was incorporated into the fiber core via emulsion electrospinning, it is necessary to assess the antibacterial properties to examine whether the functionality of cinnamon oil remains in the final material. The specimens used in the evaluation were nanocomposite fibrous membranes prepared from emulsion C (containing 12 wt% of PVA, 4.29 wt% of cinnamon oil, and 0.86 wt% of surfactant). Specimens were prepared with a web area density of 9.0 g/m^2^. After electrospinning, the membranes were dried in a vacuum oven at 50 °C for 24 h and then heat-treated at 160 °C for 1 min to stabilize PVA-based nanofibrous membranes against dissolution in a buffer solution during the antibacterial evaluation. The antibacterial assessment was carried out for the following two bacteria, *Staphylococcus aureus* (as a representative Gram-positive bacterium) and *Klebsiella pneumoniae* (as a representative Gram-negative bacterium). 

Table 5 presents the antibacterial activities of the core/sheath-structured composite nanofibrous membranes containing cinnamon oil. The nanofibrous membranes fabricated from emulsion C (containing 4.29 wt% of cinnamon oil) exhibited 99.9% reduction rates against *Staphylococcus aureus* (Gram-positive bacterium), which demonstrates that the cinnamon oil incorporated in the fiber core provided strong antibacterial effects against *Staphylococcus aureus* at the given oil concentration and that the inherent antibacterial property of cinnamon oil is retained after being embedded in the nanofibers. On the other hand, the same system did not show an inhibitory effect against *Klebsiella pneumoniae* (Gram-negative bacterium). This may be due to the more complex cell wall structure of Gram-negative bacteria. Compared with Gram-positive bacteria consisting of a single layer of peptidoglycan, Gram-negative bacteria have two layers of peptidoglycan and lipid proteins in the cell wall, which may protect these organisms [31].

### 3.4. Antifungal Properties of Core/Sheath-Structured Nanofibers Containing Cinnamon Oil

The antifungal effects of nanofibrous membranes containing cinnamon oil were assessed to investigate whether the cinnamon oil-loaded nanocomposite fibers could be used in home textiles to suppress the growth of fungi commonly found in the home. The antifungal assessment was carried out on a series of fungi that trigger respiratory- and skin-related diseases, including *Aspergillus brasiliensis* (ATCC 9642), *Penicillium funiculosum* (ATCC 11797), *Chaetomium globosum* (ATCC 6205), *Blue fungus Trichoderma virens* (ATCC 9645), and *Aureobasidium pullulans* (ATCC 15233). As shown in Table 3, the grading scale was determined by visual observation of the fungal growth. The specimens used in the assessment were composite nanofibrous membranes prepared from emulsion C (containing 12 wt% of PVA, 4.29 wt% of cinnamon oil, and 0.86 wt% of surfactant). Prior to the assessment, the membranes were dried in a vacuum oven at 50 °C for 24 h and then heat-treated at 160 °C for 1 min.

**Figure 8:**
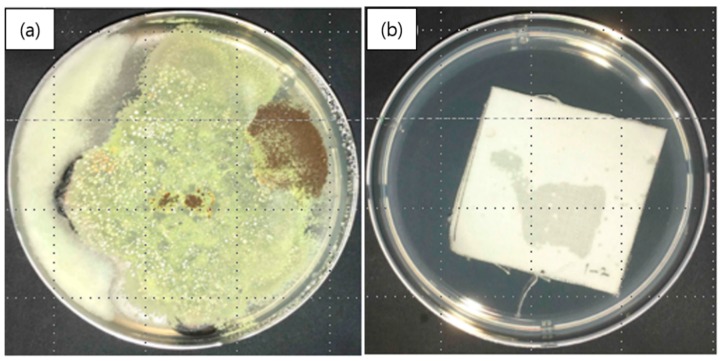
Antifungal activity of nanofibrous membranes containing cinnamon oil: (**a**) control specimen; and (**b**) nanofibers containing cinnamon oil.

Figure 8 shows a comparison between fungal growth on a control specimen (Figure 8a) and on a nanofibrous membrane containing cinnamon oil (Figure 8b) in a medium inoculated with the five fungal species after culturing for 28 days. Figure 8a shows that the fungal growth on the control specimen was so large as to cover the entire petri dish containing the strain-inoculated medium. In contrast, Figure 8b clearly shows that there was no fungal growth observed after contact with the nanocomposite fibers containing cinnamon oil. The grading was determined to be 0, indicating strong antifungal effects against the selected fungal species. These findings indicate that the cinnamon oil encapsulated in the nanofibers was released continuously for 28 days and was able to exert its antifungal effects even after it had been subjected to drying and heat treatment. It is assumed that the PVA sheath of the fiber acted to prevent the loss of volatile organic compounds (VOCs) in cinnamon oil to some extent, resulting in a sustained release of cinnamon oil over 28 days.

### 3.5. Release Behavior of Cinnamon Oil Emitted from Composite Nanofibers

The release profile of cinnamon oil from the nanocomposite fibers was assessed over a 28-day period to find out if the cinnamon oil was released in a sustainable manner from the core of composite fibers. Prior to assessing the release behavior of cinnamon oil from the composite fibers, the composition of VOCs emitted from cinnamon bark oil used in this study was analyzed by TDS-GC/MS under the same conditions used for the composite fibers. According to the GC-MS analysis, cinnamaldehyde (38.78%) was the major component, followed by eugenol (9.98%) and caryophyllene (8.18%). Singh et al. [32] also reported cinnamaldehyde as the most emitted component from their cinnamon bark volatile oil. As for monoterpenoids, cinnamaldehyde was found to be the most effective acaricidal constituent of essential oils [32]. In addition, linalool (3.46%), eucalyptol (2.32%), and *p*-cymene (0.94%) were released from our cinnamon bark oil, and these components are also known to have acaricidal effects against house dust mites [33,34]. Studies by Sánchez-Ramos and Castañera [35] showed that monoterpene components containing oxygen, such as eucalyptol and linalool, were 98% effective against house dust mites (*Tyrophagus putrescentiae*) even in low concentrations.

The release behavior of cinnamon oil from the fibers was examined in composite fibers that had shown the acaricidal effects against house dust mites and antimicrobial properties respectively, i.e., the composite fibers prepared from emulsion H (containing 12 wt% of PVA, 20 wt% of cinnamon oil, and 4 wt% of surfactant) and emulsion C (containing 12 wt% of PVA, 4.29 wt% of cinnamon oil, and 0.86 wt% of surfactant). The release characteristics over time were examined only for the composite fibers prepared from emulsion C. The assessment was performed on the as-spun fibers without drying or heat treatment. The release characteristics of cinnamon oil from the composite fibers were assessed five times during the testing period, i.e., immediately after electrospinning and 7, 14, 21, and 28 days after electrospinning.

Table 6 presents the release profile of major chemical components (cinnamaldehyde, eugenol, and caryophyllene) emitted from the nanofibrous membranes containing cinnamon oil. The amounts of total VOCs and natural VOCs released from the fibers were also measured during the testing period. Natural VOC denotes terpene-based VOCs derived from plants. These are known to have beneficial effects on human immune functions, relieving stress and reducing blood pressure in humans and animals [36]. As shown in Table 6, the amounts of total VOC and natural VOC released from the composite nanofibers containing high concentrations of cinnamon oil (emulsion H) were much higher than those containing low concentrations of cinnamon oil (emulsion C), especially with respect to the amounts of cinnamaldehyde released. This may explain the findings that strong acaricidal effects against mites were exhibited by the composite fibers prepared from emulsion H but not by those from emulsion C. The release profile of cinnamon oil from the composite fibers over a 28-day period shows that the amounts of total VOCs and natural VOCs released decreased markedly during the initial 7 days of the testing period, followed by a gradual decrease throughout the remaining testing period. The three major chemical components (cinnamaldehyde, eugenol, and caryophyllene) exhibited a similar trend in release behavior over time; cinnamaldehyde particularly exhibited a substantial release during the first 7 days, and thereafter, the same rate of release was maintained until day 21 of the testing period. To examine whether the amount of volatile organic components released after 7 days was efficient, the antibacterial properties were further investigated over time considering the prolonged release. The antibacterial activity of the composite nanofibrous membranes was assessed after the specimens had been stored for 7 days. The composite nanofibers fabricated from emulsion C (containing 4.29 wt% of cinnamon oil) exhibited a 99.9% reduction rate against *Staphylococcus aureus* after 7 days, which confirms the sustainability of the antimicrobial properties of the specimens. Since the cinnamon oil release studies showed that the release rate of major chemical components, especially cinnamaldehyde, after 7 days was maintained at approximately the same level until 21 days, we conclude that the concentration of cinnamon oil released would be sufficient to inhibit bacterial growth for 21 days.

In addition, the release behavior of the samples on which cinnamon oil was sprayed was examined for comparison. For the oil-sprayed sample, 4991.1, 5386.7, and 1483.1 μg/m^3^ of total VOCs were released on day 0, day 1, and day 3, respectively, and then, the amount of VOC released decreased considerably. The amounts of total VOCs released on day 7 and day 21 were 315.3 and 81.8 μg/m^3^, respectively. The oil-sprayed sample showed an initial rapid release of VOCs during the first 2 days and thereafter a significant decrease, possibly due to the volatile nature of essential oil. These findings demonstrate that the oil-sprayed sample exhibited a much faster and greater release of VOCs in the beginning in comparison with the core/sheath-structured composite fibers with oil incorporated in the core, and did not maintain the sustained release over a long duration.

**Figure 9:**
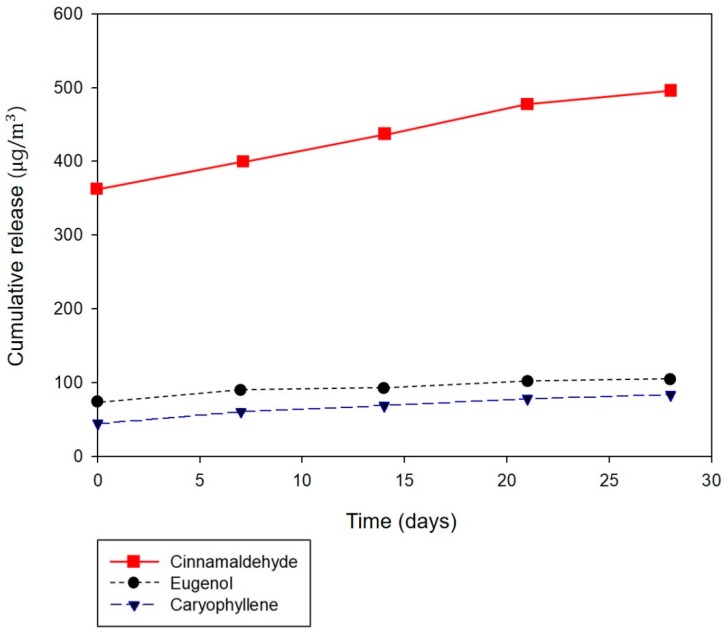
Cumulative release of the major chemical components of cinnamon oil (cinnamaldehyde, eugenol, and caryophyllene) from nanofibrous membranes containing cinnamon oil.

Figure 9 illustrates the cumulative release behavior of the three major chemical components (cinnamaldehyde, eugenol, and caryophyllene) from the composite fibers over time. Overall, the cumulative release profile of VOCs from the core/sheath-structured nanofibers containing cinnamon oil reveals a continuous release over 28 days. Although many essential oils possess various bioactivities such as antibacterial, antifungal, and antiviral properties, they must be re-applied frequently to be effective due to their volatile nature. The core/sheath-structured composite fibers in which bioactive agents were incorporated in the fiber core allowed a continuous release over a prolonged period of time. Our findings demonstrate that using such fibrous structures could be a promising approach to deliver naturally derived bioactive agents in a sustained way.

## 4. Conclusions

In this study, core/sheath-structured nanofibers containing cinnamon oil were fabricated by emulsion electrospinning, and their acaricidal effects against house dust mites as well as antibacterial and antifungal properties were examined to explore their potential applications in home textiles. To fabricate bead-free, uniform core/sheath-structured nanofibers, the most suitable emulsion formulations and processing conditions were identified. The CLSM analysis revealed a well-aligned core/sheath structure within the nanofibers. With an increase in oil concentration of up to 20 wt% in the emulsion, the core/sheath-structured nanofibers were successfully fabricated, albeit with a diameter greater than those of the nanofibers fabricated using low concentrations of cinnamon oil in the emulsion. 

Monoterpenoid compounds of cinnamon bark oils emitted from the nanofibers exhibited acaricidal effects against house dust mites as well as antibacterial and antifungal properties. Nanofibers fabricated from an emulsion containing 20 wt% of cinnamon oil achieved 100% mortality of house dust mites. Nanofibers fabricated from an emulsion containing 4.29 wt% of cinnamon oil revealed a strong antimicrobial effect against *Staphylococcus aureus* and a series of fungi but no antibacterial effect against *Klebsiella pneumoniae*. The release profile of VOCs from the core/sheath-structured nanofibers containing cinnamon oil exhibited a continuous release over 28 days. Although the amounts of VOCs released decreased considerably after 7 days, the strong antibacterial activity of the composite nanofibers against *Staphylococcus aureus* was maintained over a period of time. These results demonstrate that core/sheath-structured nanofibers containing cinnamon oil have a high potential for use in home textiles that are environmentally friendly and multifunctional.

## Figures and Tables

**Table 1:** Cinnamon oil/PVA emulsion concentrations for imparting antimicrobial activity.

Emulsion	Concentration (wt%)		
	PVA	Cinnamon Oil	Surfactant
A	10	3.57	0.71
B	11	3.93	0.79
C	12	4.29	0.86
D	13	4.64	0.93
E	14	5	1

**Table 2:** Cinnamon oil/PVA emulsion concentrations for imparting acaricidal effects against house dust mites.

Emulsion	Concentration (wt%)		
	PVA	Cinnamon Oil	Surfactant
C	12	4.29	0.86
F		8	1.6
G		15	3
H		20	4
I		30	6

**Table 3:** Grading scale for visible effects in antifungal test [19].

Observed Growth on Specimens	Rating
No growth	0
Trace of growth (less than 10% coverage)	1
Slight growth (10%–30% coverage)	2
Moderate growth (30%–60% coverage)	3
Heavy growth (60%–100% coverage)	4

**Table 4:** Acaricidal effects of nanofibrous membranes containing cinnamon oil against house dust mites at various oil concentrations.

Sample	Sample Condition	Oil Concentration in Emulsion	Acaricidal Effect (Dead/Alive)	
			Start	1 h
Nanofibrous membranes containing cinnamon oil	As-spun	4.29 wt% (emulsion C)	0/30	0/30
		8 wt% (emulsion F)		
		15 wt% (emulsion G)		
		20 wt% (emulsion H)	0/30	30/0
		30 wt% (emulsion I)		
	Dried	4.29 wt% (emulsion C)	0/30	0/30
		8 wt% (emulsion F)		
		15 wt% (emulsion G)		
		20 wt% (emulsion H)		
		30 wt% (emulsion I)		
Control	As-spun	-	0/30	0/30
	Dried	-	0/30	0/30

**Table 5:** Antibacterial effects of nanofibrous membranes containing cinnamon oil against *Staphylococcus aureus* and *Klebsiella pneumoniae.*

	Contact Time (h)	Number of Bacterial Colonies (CFU/mL)		Bacterial Reduction (%)
		Control	Nanofibrous Membranes Containing Cinnamon Oil	
*Staphylococcus aureus*	0	2.5 × 10^5^	2.5 × 10^5^	-
	24	1.3 × 10^5^	9.0 × 10	99.9
*Klebsiella pneumoniae*	0	2.1 × 10^5^	2.1 × 10^5^	-
	24	1.7 × 10^5^	3.0 × 10^5^	0

**Table 6:** Release profile of major chemical components emitted from nanofibrous membranes containing cinnamon oil.

Emulsion (Oil Concentration)	Testing Period	Total VOC ^a^ (μg/m^3^)	Natural VOC ^a^ (μg/m^3^)	Major Chemical Component			Natural VOC ^a^ (%)
				Cinnamaldehyde (μg/m^3^)	Eugenol (μg/m^3^)	Caryophyllene (μg/m^3^)	
Emulsion H (20 wt%)	Day 0	37360.0	24671.9	20063.4	7448.8	5677.4	65.9
Emulsion C (4.29 wt%)	Day 0	1379.8	461.5	362.7	73.6	44.3	35.0
	Day 7	723.2	59.5	36.3	16.0	9.9	8.8
	Day 14	489.8	68.2	37.5	2.1	8.6	13.5
	Day 21	746.0	100.4	41.3	9.6	9.0	13.1
	Day 28	291.3	32.4	18.0	3.1	5.5	11.4

^a^ VOC (volatile organic compound).
